# Efficacy and Selectivity of Potassium Bicarbonate Salts against *Cacopsylla pyri* on Pears

**DOI:** 10.3390/insects14060491

**Published:** 2023-05-24

**Authors:** Stefano Civolani, Mauro Boselli, Emanuele Radicetti, Giovanni Bernacchia

**Affiliations:** 1 Department of Chemical, Pharmaceutical and Agricultural Sciences, University of Ferrara, Via Borsari 46, 44121 Ferrara, Italy; rdcmnl@unife.it; 2 Independent Researcher in Applied Entomology, Pianoro, 40065 Bologna, Italy; boselli56@libero.it; 3 Department of Life Sciences and Biotechnology, University of Ferrara, Via Borsari 46, 44121 Ferrara, Italy

**Keywords:** inorganic salts, field trial, pear psylla control, IPM, insecticide

## Abstract

**Simple Summary:**

Potassium bicarbonate is a mineral salt used as a fungicide on different crops, such as pears. Recently, it has also appeared to have some insecticidal activity against arthropod pests. Pear orchards are usually infested by *Cacopsylla pyri* that cause serious losses. Chemical pesticides (such as spirotetramat) are still cornerstone methods to control this pest, but are soon to require substitution by more environment-friendly products. For this reason, we tested the effects of potassium bicarbonate, with or without an adjuvant, as compared to spirotetramat, on the reduction of *C. pyri* juvenile forms in two field trials. The higher rate tested (7 kg ha^−1^) appeared more active on the pest and did not show any phytotoxic effects on pear plants. Potassium bicarbonate could, therefore, represent a viable product in the control of pear psylla and it might be usable in integrated management strategies focused on other plant pathogens, such as *Stemphylium vesicarium*.

**Abstract:**

In recent years, the control of pear psyllid in northern Italy has not been particularly problematic, due to the presence of two insecticides (abamectin and spirotetramat) specifically for this pest, and due to the adoption of integrated pest management. However, the withdrawal of these two specific insecticides is imminent and, therefore, it has become necessary to find alternative control tools. More recently, potassium bicarbonate, known for its fungistatic activity against many phytopathogenic fungi, has also shown some activity against some insect pests. In the present study, the efficacy and possible phytotoxicity of potassium bicarbonate were tested in two field trials on second generation *Cacopsylla pyri* by spraying two different salt concentrations (5 and 7 kg ha^−1^), with or without polyethylene glycol as an adjuvant. Spirotetramat was used as a commercial reference. The results showed that potassium bicarbonate could positively control the number of juvenile forms (with a mortality percentage of up to 89% at the infestation peak), even though spirotetramat was still more effective. Therefore, potassium bicarbonate appears to be a sustainable integrated tool for psyllid control, especially in the wake of the imminent withdrawal of spirotetramat and other insecticides currently used on this pest.

## 1. Introduction

The pear psyllids (Hemiptera: Psylloidea: *Cacopsylla*) are small specialist insects sap-feeding on pears. Their feeding habits cause russet on, and downgrading of, the fruit, due to marking of the fruit by honeydew and sooty mold, premature leaf drop and tree weakening in commercial pear orchards. Furthermore, they are vectors for a specific pear disease called “pear decline”, caused by the phytoplasma *Candidatus* Phytoplasma pyri. The economic loss caused by pear psyllids increased noticeably after the second World War because of intensive pear cultivation, use of synthetic pesticides in orchards, and pesticide resistance. Among the most harmful and widespread species are the following: *Cacopsylla chinensis* (Yang and Li), present in the Far East and especially in China; *Cacopsylla bidens* (Šulc) in the Middle East and introduced into Latin America; *Cacopsylla pyricola* (Foerster), widespread in northern Europe and introduced into North America; *Cacopsylla pyri* L., mostly present in southern Europe and uniquely present in the pear orchards of Italy [1]. In this country, and in particular in the Emilia-Romagna region (northern Italy), over the past two decades, *C. pyri* has generally caused less damage to pear orchards thanks to successful integrated pest management programs (IPMs). These programs are based on the use of a limited number of specific pesticides, such as abamectin and spirotetramat [2,3], unlike in many other pear growing areas, such as in the United States, where a higher number of insecticides are used. Spirotetramat, the most recent to be introduced in Italy, is a tetramic acid derivative [3], highly effective against *C. pyri* and representing a good alternative to the older abamectin, introduced in 1996 [1,2].

Unfortunately, the European Union has confirmed the expiration of approval for spirotetramat in 2024 [Regulation EU 2022/489], while the approval expiration date for abamectin is under review [EU 2023/515]. The search for new sustainable insecticides to control psyllids has been instigated by these regulatory developments. Further incentives for the search arise from the fact that pear breeding has not yet been able to release Psylla-resistant cultivars [4,5], and sexual pheromones-based strategies have not been developed for this pest [6]. Furthermore, pear cultivation in Italy has undergone a significant decline in terms of production due to a series of biotic stresses that have brought down this important agricultural sector in northern Italy, the worst of these being the invasion of *Halyomorpha halys* and the spread of brown spot, and *Stemphylium vesicarium*. Since 2018, the latter has become more virulent, especially in the cultivar Abbè Fétel. To control brown spot, synthetic fungicides are usually employed. Recently, an alternative strategy has been applied, based on the use of mineral salts [7]. Among these, potassium bicarbonate is mostly known as an inorganic fungicide (with fungistatic activity [8]), registered for use in agricultural and greenhouse cropping systems for the control of various plant pathogens [9,10,11,12,13], including *S. vesicarium* (S. Civolani, based on personal communication). Even though the effects of this fungicide have not been properly evaluated against insect pests, some activity has been observed in regard to citrus mealybug, *Planococcus citri* [14]. The aim of this study was to test the efficacy of two different concentrations of potassium bicarbonate, applied with or without adjuvant, against *C. pyri* in pear orchards, to assess it as a possible substitute for the soon to be banned pesticides commonly used against *C. pyri*.

## 2. Materials and Methods

### 2.1. Insecticides

Potassium bicarbonate (85% SP) and Polyethylene glycol PEG 400 were supplied by Ubuntu Gmbh, Baar, Switzerland, and Spirotetramat (Movento 48 SC), by Bayer CropScience, Milan, Italy

### 2.2. Experimental Conditions

**Figure 1:**
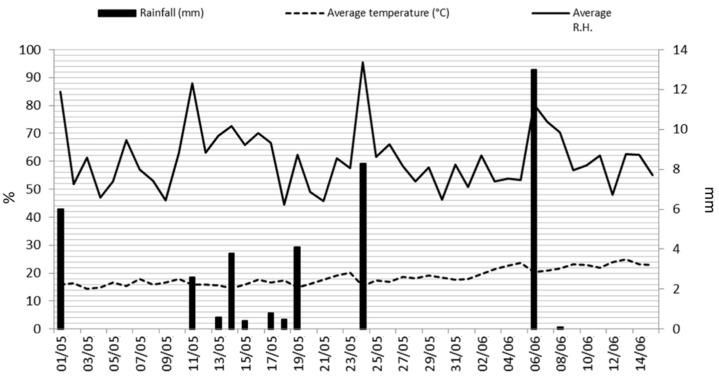
Meteorological data during the field trials.

The efficacy of potassium bicarbonate in the control of the most aggressive generation of *C. pyri* (the second generation, which is usually present in Italy in May) was evaluated in two field trials, in 2021, in two different commercial pear orchards located in Ferrara province (Emilia-Romagna Region, Italy). The first field trial was on cv. Abbè Fètèl with vigorous shoots (GPS coordinates: 44.796163, 11.709860), and the second one was on cv. Kaiser Alexander with very vigorous shoots (GPS coordinates: 44.465548, 11.360300). The field trials were carried out according to Good Experimental Practice (GEP) and following the EPPO/OEPP guidelines (https://archives.eppo.int/EPPOStandards/efficacy.htm, accessed on 4 April 2021), based on protocol number PP 1/44 (2). The experimental design was a randomized complete block (RCB) with four replications per treatment. In the first trial, each plot measured 29.4 m^2^ (8.4 × 3.5 m, 7 trees), while in the second trial each plot measured 22.75 m^2^ (7 × 3.25 m, 7 trees). Potassium bicarbonate was dissolved in water at two concentrations (5 and 7 kg ha^−1^), with or without polyethylene glycol as the adjuvant (1 L ha^−1^). Spirotetramat was applied at 4.5 L ha^−1^, while polyethylene glycol was applied at 1 L ha^−1^ alone as a further control. The untreated control plot was not subjected to any spraying. The solutions were applied with an application volume of 1000 L ha^−1^ with a mist blower knapsack sprayer (Stihl sr 430). The applications in both trials were performed on 14 May 2021, on the yellow eggs *C. pyri* stage, and were repeated on 28 May 2021. The levels of pest infestation in the different treatments were assessed four times weekly as the number of juvenile forms (the sum of neanids and nymphs per shoot, which are the most damaging forms in pear orchards), on 10 pre-marked shoots per plot, each homogenously populated with *C. pyri* eggs (chosen the day before the applications). Meteorological data during the trial periods are depicted in Figure 1.

### 2.3. Statistical Analysis

The mean number of juvenile psylla per shoot (neanids and nymphs taken together, on 10 shoots per plot) was compared across treatments by using ANOVA, followed by the Newman–Keuls test for post-hoc comparison of means. All analyses were performed by using STATISTICA 6 software. The efficacy of each treatment in respect to the untreated control was calculated using the Abbott formula [15].

## 3. Results

Table 1 and Table 2 describe the efficacy of the application of potassium bicarbonate, alone or in combination with polyethylene glycol (PEG) as the adjuvant, compared to the specific insecticide, spirotetramat, in two field trials.

In the first field trial, at the time of the first application (14 May 2021), in each plot there were *C. pyri* adults, mature eggs and some young neanids. The first assessment (see Table 1) showed an abundant infestation in the untreated control (27.9 juvenile individuals per shoot, no nymphs). The most significant reduction of the infestation (93% efficacy) was observed with spirotetramat. Potassium bicarbonate (alone or in a tank mix with PEG) showed noteworthy efficacy (ranging between 63 and 73%), as compared to the untreated control, but no statistically significant difference was observed between the two different concentrations of potassium bicarbonate (5 or 7 kg ha^−1^). PEG alone showed significant, but lower, efficacy (31.9%) in respect to the untreated control.

The second weekly assessment (28 May 2021, Table 1) was performed just before the second application. The natural infestation increased, as expected, in the untreated control, showing a mixed presence of *C. pyri* neanids and nymphs (63.3 juvenile individuals per shoot). The most significant reduction of the infestation (97% efficacy) was again observed for spirotetramat, while both concentrations of potassium bicarbonate, mixed with PEG, showed significant moderate efficacy (42.5% and 67.9%). Potassium bicarbonate alone had lower efficacy (35.2%) as compared to the mixtures with PEG. The adjuvant alone showed no significant difference in respect to the untreated control.

The third weekly assessment in the untreated control (Table 1, 4 June 2021), performed one week after the second application, revealed, as expected, a moderate natural decrease of juvenile individuals. Therefore, the infestation appeared lower (42.5 juvenile individuals per shoot). In this scenario, spirotetramat maintained high efficacy. The efficacies of both concentrations of potassium bicarbonate, mixed with PEG, showed significant increase (76.7% and 89.4%), as compared to the previous assessment, and were almost comparable to spirotetramat. Moderate efficacy (66.1%) was observed for the potassium bicarbonate alone, while the adjuvant alone (PEG) was still comparable to the untreated control.

The last assessment was performed two weeks after the second application (11 June 2021, Table 1) and revealed a remarkable decrease in natural infestation by juvenile individuals, as observed against the untreated control (16.4 juvenile individuals per shoot). Even though this terminal assessment was less noteworthy, spirotetramat maintained high efficacy, as did both concentrations of potassium bicarbonate, with or without PEG. The efficacy of PEG alone was again comparable to the untreated control.

The second field trial was conducted in an old pear orchard (cv. Kaiser Alexander) with very vigorous shoots, in the presence of the same *C. pyri* forms as those of the first field trial and on the same dates.

The first assessment (see Table 2) showed an abundant infestation in the untreated control (28.8 juvenile individuals per shoot, no nymphs). The most significant reduction of the infestation (84.4% efficacy) was observed with spirotetramat, closely followed by the high concentration of potassium bicarbonate (74.7% and 76.7%) independently of the adjuvant PEG. Potassium bicarbonate at a lower concentration, mixed with PEG, showed lower efficacy (52.4%). No significant difference was observed for PEG alone in respect to the untreated control.

The second assessment (Table 2), performed just before the second application, revealed a natural increase of the infestation, as observed in the untreated control, that showed a mixed presence of *C. pyri* neanids and nymphs (81.4 juvenile individuals per shoot). The most significant reduction of the infestation (97.3% efficacy) was again observed for spirotetramat, while significant moderate efficacy (nearly 50%) was observed for all the potassium bicarbonate concentrations, with or without PEG. No significant difference was observed with PEG alone in respect to the untreated control.

The third assessment was performed one week after the second application and showed a moderate natural decrease of infestation (52.6 juvenile individuals per shoot), as observed in the untreated control (Table 2). In this condition, spirotetramat maintained high efficacy (99%), while the efficacies of all potassium bicarbonate treatments appeared lower, even though there was no statistical difference (78.3%, 84.8% and 86.1%).

The final assessment (Table 2), was less noteworthy. as in the first trial, showed high efficacy for spirotetramat. as well as for both of potassium bicarbonate, with or without PEG. The efficacy of PEG alone was again comparable to the untreated control. No phytotoxic effects were observed in any plots in the two field trials.

## 4. Discussion and Conclusions

Two field trials tested the possible use, on pear plants, of the fungicide potassium bicarbonate (an inorganic salt with known fungistatic activity) for the control of pear psylla. This salt might act as an environment-friendly alternative to chemical insecticides, such as spirotetramat (the approval of which expires soon). The salt was tested at two different concentrations, with or without an adjuvant, PEG. The weekly assessments indicated that, in the case of high infestation of *C. pyri*, potassium bicarbonate, when applied in high concentration (7 kg ha^−1^), could satisfactorily control the spread of the pest in the orchard, when compared to the high efficacy of spirotetramat as a reference product. Two potassium bicarbonate applications appeared to achieve better results, maybe because the second application synchronized with the peak in presence of different *C. pyri* juvenile individuals. The tank mix with an adjuvant, PEG (1 L ha^−1^), did not significantly improve the efficacy of the potassium bicarbonate salts, and, therefore, its role remains uncertain. The trial also showed that the adjuvant PEG applied alone had no significant efficacy against the pest.

These preliminary data indicate that this known fungicide might also be used as an insecticide even at high rates of application with no phytotoxic effects on pear plants. This is one of the few records of the potential use of potassium bicarbonate as an insecticide, alongside its known fungicide activities in pears and other crops [9,10,11,12,13]. The only reported evidence thus far has concerned coleus plants, *Solenostemon scutellarioides*, infested by the citrus mealybug, *Planococcus citri* [14]. Similar salt rates provided comparable efficacy to our data, while higher rates (14.9 kg ha^−1^) induced phytotoxicity on this ornamental plant. Despite some information on the mechanism of action on pathogenic fungi [8], there is no information available on the possible mechanism of action on pest insects.

This salt might, therefore. act as an environment-friendly alternative to chemical insecticides, such as spirotetramat (the approval of which expires soon). Further investigations will help define the best way to couple the potential use of this fungicide in systematic spraying against pear brown spot, caused by *S. vesicarium*, with its insecticidal activity against *C. pyri*. This integrated approach would fit with present and future IPM programs, such as the EU Farm to fork directive [16], based on environment-friendly non-synthetic insecticides.

## Figures and Tables

**Table 1:** Efficacy of the different treatments in the first field trial: mean number of juvenile individuals (neanids and nymphs taken together) per shoot.

	Mean Number of Juvenile Individuals per Shoot							
	21 May		28 May		4 June		11 June	
Treatment	Mean ± SE *	Eff. % **	Mean ± SE *	Eff. % **	Mean ± SE *	Eff. % **	Mean ± SE *	Eff. % **
Untreated Control	27.9 ± 2.97 a		63.3 ± 5.32 a		42.5 ± 3.59 a		16.4 ± 2.40 a	
Polyethylene glycol 1 L ha^−1^	19.0 ± 2.78 b	31.9	48.9 ± 6.22 ab	22.7	41.1 ± 6.58 a	3.3	13.1 ± 1.38 a	20.0
Potassium bicarbonate 7 kg ha^−1^	10.2 ± 1.20 c	63.4	41.0 ± 6.23 b	35.2	14.4 ± 2.04 b	66.1	1.4 ± 0.67 b	91.4
Potassium Bicarbonate 5 kg ha^−1^ Polyethylene glycol 1 L ha^−1^	9.9 ± 0.98 c	64.5	36.4 ± 6.02 c	42.5	9.9 ± 0.98 bc	76.7	2.3 ± 0.87 b	86.0
Potassium bicarbonate 7 kg ha^−1^ Polyethylene glycol 1 L ha^−1^	7.0 ± 0.78 c	74.9	20.3 ± 4.17 c	67.9	4.5 ± 1.33 bc	89.4	1.1 ± 0.41 b	93.3
spirotetramat 4.5 L ha^−1^	1.7 ± 0.71 d	93.9	1.9 ± 0.91 d	97.0	1.4 ± 0.30 c	96.7	0.3 ± 0.13 b	98.2

(*) Values marked by different letters are statistically different (NK Test *p* ≤ 0.05). *n* = 4 plots with 10 shoots each. (**) Efficacy obtained by Abbott formula.

**Table 2:** Efficacy of the different treatments in the second field trial: mean number of juvenile individuals (neanids and nymphs taken together) per shoot.

	Mean Number of Juvenile Individuals per Shoot							
	21 May		28 May		4 June		11 June	
Treatment	Mean ± SE *	Eff. % **	Mean ± SE *	Eff. % **	Mean ± SE *	Eff. % **	Mean ± SE *	Eff. % **
Untreated Control	28.8 ± 0.91 a		81.4 ± 12.74 a		52.6 ± 4.17 a		16.5 ± 2.54 a	
Polyethylene glycol 1 L ha^−1^	28.0 ± 2.57 a	2.8	70.2 ± 5.46 a	13.6	45.1 ± 5.84 a	14.3	7.9 ± 0.77 b	52.1
Potassium bicarbonate 7 kg ha^−1^	7.3 ± 2.29 c	74.7	38.9 ± 3.44 b	52.1	11.4 ± 2.56 b	78.3	4.5 ± 1.27 bc	72.7
Potassium Bicarbonate 5 kg ha^−1^ Polyethylene glycol 1 L ha^−1^	13.7 ± 1.87 b	52.4	42.3 ± 3.81 b	48.0	8.0 ± 1.38 b	84.8	0.7 ± 0.18 c	95.8
Potassium bicarbonate 7 kg ha^−1^ Polyethylene glycol 1 L ha^−1^	6.7 ± 1.04 c	76.7	38.4 ± 1.29 b	52.8	7.3 ± 1.45 b	86.1	2.1 ± 0.64 c	87.3
spirotetramat 4.5 L ha^−1^	4.5 ± 1.41 c	84.4	2.2 ± 1.44 c	97.3	0.5 ± 0.23 b	99.0	0.1 ± 0.09 c	99.4

(*) Values marked by different letters are statistically different (SNK Test *p* ≤ 0.05). *n* = 4 plots with 10 shoots each. (**) Efficacy obtained by Abbott formula.

## Data Availability

The datasets generated and analyzed during the current study are available within the article, as well as from the corresponding author on reasonable request.

## References

[1] Civolani S., Boselli M., Butturini A., Chicca M., Cassanelli S., Tommasini M.G., Aschonitis V., Fano E.A. (2015). Testing Spirotetramat as an Alternative Solution to Abamectin for *Cacopsylla pyri* (Hemiptera: Psyllidae) Control: Laboratory and Field Tests. J. Econ. Entomol..

[2] Civolani S., Cassanelli S., Rivi M., Manicardi G.C., Peretto R., Chicca M., Pasqualini E., Leis M. (2010). Survey of susceptibility to abamectin of pear psylla (Hemiptera: Psyllidae) in northern Italy. J. Econ. Entomol..

[3] Brück E., Elbert A., Fischer R., Krueger S., Kühnhold J., Klueken A.M., Nauen R., Niebes J.F., Reckmann U., Schnorbach H.J. (2009). Movento®, an innovative ambimobile insecticide for sucking insect pest control in agriculture: Biological profile and field performance. Crop Prot..

[4] Nin S., Ferri A., Sacchetti P., Giordani E. (2012). Pear resistance to Psilla (*Cacopsylla pyri* L.). A review. Adv. Hortic. Sci..

[5] Dondini L., De Franceschi P., Ancarani V., Civolani S., Fano E.A., Musacchi S. (2015). Identification of a QTL for psylla resistance in pear via genome scanning approach. Sci. Hortic..

[6] Ganassi S., Germinara G.S., Pati S., Civolani S., Cassanelli S., Sabatini M.A., De Cristofaro A. (2018). Evidence of a female-produced sex pheromone in the European pear psylla, *Cacopsylla pyri*. Bull. Insectology.

[7] Khursheed A., Rather M.A., Jain V., Wani A.R., Rasool S., Nazir R., Malik N.A., Majid S.A. (2022). Plant based natural products as potential ecofriendly and safer biopesticides: A comprehensive overview of their advantages over conventional pesticides, limitations and regulatory aspects. Microb. Pathog..

[8] Fallir E., Grinberg S., Ziv O. (1997). Potassium bicarbonate reduces postharvest decay development on bell pepper fruits. J. Hortic. Sci..

[9] Möth S., Redl M., Winter S., Hüttner F., Steinkellneret S. (2023). Efficiency of inorganic fungicides against the formation of *Erysiphe necator* chasmothecia in vineyards. Pest Manag. Sci..

[10] Abd-El-Kareem F. (2007). Potassium or sodium bicarbonate in combination with Nerol for controlling early blight disease of potato plants under laboratory, greenhouse and field conditions. Egypt. J. Phytopathol..

[11] Cushman K.E., Evans W.B., Ingram D.M., Gerard P.D., Straw R.A., Canaday C.H., Wyatt J.E., Kenty M.M. (2007). Reduced foliar disease and increased yield of pumpkin regardless of management approach or fungicide combinations. HortTechnology.

[12] Ziv O., Zitter T.A. (1992). Effects of bicarbonates and film-forming polymers on cucurbit foliar diseases. Plant Dis..

[13] Ziv O., Hagiladi A. (1993). Controlling powdery mildew in euonymus with polymer coatings and bicarbonate solutions. HortScience.

[14] Hogendorp B.K., Cloyd R.A. (2013). Effect of Potassium Bicarbonate (MilStop) and Insecticides on the Citrus Mealybug, *Planococcus citri* (Risso), and the Natural Enemies *Leptomastix dactylopii* (Howard) and *Cryptolaemus montrouzieri* (Mulsant). HortScience.

[15] Abbott W.S.A. (1925). Method for computing the effectiveness of an insecticide. J. Econ. Entomol..

[16] Farm to Fork Strategy. https://food.ec.europa.eu/horizontal-topics/farm-fork-strategy_en.

